# Sub-Saharan African immigrant women's experiences of (lack of) access to appropriate healthcare in the public health system in the Basque Country, Spain

**DOI:** 10.1186/s12939-019-0958-6

**Published:** 2019-04-24

**Authors:** Iratxe Pérez-Urdiales, Isabel Goicolea, Miguel San Sebastián, Amaia Irazusta, Ida Linander

**Affiliations:** 1 Department of Nursing I, Faculty of Medicine and Nursing, University of the Basque Country (UPV/EHU), Barrio Sarriena S/N Leioa, Biscay, Spain; 2 Department of Public Health and Clinical Medicine, Epidemiology and Global Health, Umeå University, Umeå, Sweden

**Keywords:** Health access, Immigrant health, Health services research, Health disparities, Barriers to healthcare, Women’s health, Undocumented immigrants, Illegal immigrants, Immigration, Qualitative research

## Abstract

**Background:**

Immigrant populations face diverse barriers to accessing appropriate healthcare services on several levels. In the Basque Country, Sub-Saharan African women were identified as facing the largest barriers to access them. The aim of the study is to analyse Sub-Saharan African immigrant women's perceptions and experiences of access to appropriate healthcare in the public health system in the Basque Country, Spain.

**Methods:**

Fourteen women from eight Sub-Saharan African countries who have used the Basque public healthcare services were interviewed. A qualitative content analysis was applied: meaning that units were identified, coded and the resulting codes were then organized into three categories.

**Results:**

The first category, *Fearing to enter a health system perceived as not friendly for immigrants,* included factors, mainly those related to legal conditions for accessing healthcare services and lack of lawful documentation, that made women avoid or discontinue seeking out healthcare.

The second category, *Being attended on professionals' own communication terms,* comprised how the lack of effective communication compromised not only the access of the immigrant women to healthcare services, but also their health.

Lastly, the third category, *Is mistreatment based on racism or merely on bad luck?* described how being an immigrant and black influenced the way they were (mis)treated in the health system.

**Conclusion:**

For Sub-Saharan African immigrant women, accessing appropriate healthcare in the Basque Country was perceived to be subject to institutional barriers. At the legal level, barriers included lack of entitlement, difficulties in fulfilling legal access conditions and lack of documentation. The lack of communication with health centre staff and their attitudes, guided by a stereotyped social image of immigrants and black people, also hindered their possibilities of receiving appropriate healthcare. Facilitators for accessing healthcare included strategies from individual professionals, personal networks and social actors to help them to cope with the barriers. There is a need of reinforcing inclusion values and rights-based approach to attention among staff at the health centres to have more non-discriminatory and culturally appropriate health systems.

## Background

Immigrant populations face diverse barriers to access healthcare on several levels, mainly the legal and policy arena, the healthcare system organization, and professionals' behaviour [1, 2]. From a person-centred perspective, access to healthcare is defined as “*the opportunity to reach and obtain appropriate health care services in situations of perceived need for care*”, in which appropriateness denotes the fit between provided services and patients' needs [3]. For immigrants, to receive an appropriate healthcare is also related to the way their social and cultural differences are respected and considered in the professional-patient relationship [4].

European countries have established different legal conditions for natives and immigrants and asylum seekers to access healthcare [5, 6]. Undocumented immigrants, usually coming from low-income countries, have even more restricted legal healthcare access [7]. Due to their social vulnerability and poorer living conditions, barriers such as lack of awareness about their rights, fear of being reported to the police and poor knowledge of the local language hinder their healthcare access [8, 9]. Among immigrants, women are more likely to suffer mental and sexual and reproductive health issues than their male counterparts [10, 11], making them to interact more with healthcare professionals. They are also more frequently exposed to gender-based violence during their migration process [12].

Among the barriers that hinder access of immigrants to healthcare services, structural racism, referring to *"ideologies, practices, processes and institutions that operate to produce and reproduce differential access to power and to life opportunities along racial and ethnic lines, creating ‘undesirable others’ or ‘threats of a nation’* [13] is commonly found. Racism as a social determinant of health and its negative influence on mental and physical health outcomes has been widely studied, especially in the USA setting [14, 15]. Studies have, for example, stated that a reason that contributes to black people's worse health outcomes may be clinicians' racial biases on diagnosing and treating their health problems [16]. In Europe, a study from England identified that black and minority ethnic communities were more likely to have poorer health outcomes, more difficulty in accessing healthcare and reported having felt being given less treatment opportunities and received more inadequate treatment [17]. In France, immigrant women original from Africa, former French colonies and Turkey, were two to four times more likely to have been mistreated by caregivers in comparison with natives, with their origin and skin colour being the main explanatory factors [18]. In general, how structural racism influence healthcare access has been mainly studied through individual caregivers' practices [19], while other ideological and institutional dimensions have not been deeply considered.

Professionals at the health centres contribute to preserve given power relations through their actions, carried out in routine ways according to accepted cultural values and social patterns [19]. However, they do not always intentionally provide a differentiated attention to patients on the basis of their cultural and social position [20]. Therefore, racist practices by healthcare staff that take place in everyday practising remain often invisible [18]. Health systems in relation to structural racism are not based on fixed dynamics, as healthcare professionals' good practices can not only influence how individual care-seekers are treated, but also contribute to transform the organization of the system.

Existing data on access to healthcare for immigrants in the Spanish context show that in general they use less healthcare services and have better health outcomes [21, 22]. However, studies analysing the actual experiences of immigrants, particularly women, are scarce. Existing studies have shown that African women have worse compliance with recommended consultations and tests in prenatal care than natives [23] and worse adherence to HIV related medical treatment [12]. Reasons given for lack of adherence, do not differ from those that hinder immigrants' access to healthcare, such as insufficient communication skills in the local language, administrative barriers, stigma and discrimination, different health beliefs and habits and social risk factors [12, 23–25].

In the Basque Country, which is one of the 17 autonomous regions in which Spain is divided, a study from 2008 showed that the utilisation of healthcare services by immigrants was higher among women [26]. In the same setting, immigrant women from Sub-Saharan Africa were identified as facing the largest barriers to access the public healthcare services in comparison to other origin immigrant women [2]. Interpreting the experiences “in context” can shed light on how historical and social relations influenced by racialization, gender and migration status interact to organize individual healthcare experiences [27]. Considering these facts, the objective of the study was to analyse Sub-Saharan African immigrant women's perceptions and experiences of access to appropriate healthcare in the public health system in the Basque Country, Spain.

## Methods

### Study context

In the Basque Country, by January 2018, 9.4% of the population was represented by registered immigrants, namely 151,128 people, of which near half of them were women and 7.7% were Sub-Saharan Africans [28]. In a legal document of December 2017, the amount of undocumented immigrants in the autonomous region was estimated between 14,632 and 22,886 [29].

Each autonomous region in Spain has its own public health system, ruled by same state laws but different regional norms [30]. Until 2012, Spain had one of the most inclusive laws in regards to access to healthcare for undocumented immigrants and asylum seekers among the European countries. Only three months of registration in any Spanish municipality was required to get the healthcare card [31], which is the document that entitles individuals to healthcare access [32]. However, the Spanish government dramatically reduced the access to healthcare services for undocumented immigrants in 2012 [7]. In the same year, the Basque Country government launched the regional Decree 114/2012 changing from three to twelve months of consecutive municipality registration the requirement to access healthcare services [33]. However, after this study was conducted, a new general state law was approved in July 2018, restoring the conditions in force before the 2012 law reform [34].

The way to enter the health system, in case of not having an emergency, is through primary healthcare centres. Healthcare is only provided prior appointment, given by administrative staff, who are also responsible for providing information and applying the legal norms and consequently, to allow or deny receiving healthcare services [35].

Likewise, to ensure access to healthcare for underserved populations, a free clinic providing no-cost primary health support exists in Bilbao, the city with the highest number of immigrants in the autonomous region. Most of the patients are undocumented immigrants, as for receiving attention, there is need of not fulfilling the conditions for access to the public healthcare system. Other social organisations also work with immigrants to increase their information about their rights, develop skill-building activities and promote their gathering and empowerment.

### Data collection

The first author collected the data from June 2016 to October 2017. The recruitment was made in different ways: First, Sub-Saharan African women attending a free clinic were directly invited to participate while they waited for being attended in the medical consultation. Secondly, social organizations were contacted for recruiting participants among their users. In a final stage, Sub-Saharan African women working at contacted social organizations were also invited to participate.

The participants were purposively selected based on the following criteria: being an immigrant woman from any Sub-Saharan African country, living in the Basque Country and having used the regional public healthcare services on at least one occasion. In total, fourteen women from eight different countries were interviewed. Among them, four were undocumented by the time of the interview and from the ten documented, four had been undocumented before. Sociodemographic characteristics of the participants are described in Table 1.

**Table 1:** Characteristics of the participants

Migration status	Documented (10) of which have been undocumented before (4)	Undocumented (4)
Country of origin	Cameroon Guinea Bissau (2) Senegal Angola (3) Central African Republic Democratic Republic of Congo Gambia	Democratic Republic of Congo (2) Nigeria Cameroon
Type of residence permit	Regrouped by husband (4) Asylum-seeker (2) Others (4)	None
Use of Public Health System	Whenever it was needed	1 time (1) 2 times (2) 3 times (1)
Use of other health services	Free clinic before having Health Card (2) Private health services (1)	Free clinic (4)
Possession of Health Card	10	None
Age	25 to 35 (3) 36 to 60 (6) More than 60 (1)	25 to 35 (3) 36 to 60 (1)
Language used in the interview	Spanish (9) French (1)	French (2) English (1) Swahili (with translator) (1)
Time in the Basque Country by the day of the interview	Less than 6 months (1) 6 months-1 year (1) 1–5 years (2) More than 5 years (6)	Less than 6 months (2) 6 months-1 year (2) 1–5 years More than 5 years
Job	Formal job (6)	None

Nine interviews were made in Spanish, one in English, three in French and one in Swahili and French. In two occasions, based on the preference of the participants, translators from French to Spanish were used. In one case, a translator was used from Swahili to French. All supporting translators were provided by the participants.

A semi-structured guide was used that was modified during the data collection process to discuss emergent interesting topics during subsequent interviews. All interviews started with an open-ended question about their experience with the Basque public health system. Questions regarding the influence of the legal norms, language, migrant status and other factors in access to health care were also posed. Finally, the interviewer explored participants' opinions and experiences regarding racist attitudes in the society and within the healthcare system. Interviews lasted between 25 to 70 min. Data collection continued until no remarkable additional information related to the aim appeared in the last interviews compared to the previous interviews.

### Data analysis

All the interviews were transcribed and reviewed for accuracy by the first author. The interviews held in English and Spanish were transcribed verbatim, while the interviews conducted in French were translated into Spanish for analysis. All the transcripts were afterwards entered into the *Open Code 4.03 Qualitative data analysis* software to facilitate organizing and coding the data. Data were analysed using qualitative content analysis, which implies the systematic description of the manifest content and the interpretation of the latent content of the text. Analysis was developed following the approach proposed by Graneheim and Lundman, focusing on the manifest content [36]. The analysis began with the first two authors carefully reading the transcripts in order to become familiar with the content. Then, parts of the text relating to the research question were identified, representing the meaning units. Meaning units were coded line by line and codes were grouped in four preliminary categories. Once all the data was organized, preliminary results were discussed between all authors, the codes were re-organized and three categories were developed.

### Ethical considerations

During data collection, all participants were given written and oral information about the goals of the study and gave written consent for participating and being audio recorded during the interview.

Differences in power relations between researcher and participants may have influenced the willingness to participate. The author who collected the data presented herself as a researcher at the regional university and a nurse volunteering at the free clinic when asking the women to participate. The free clinic and the social organisations where participants were recruited are considered immigrant-friendly, which presumably increased the trust of the immigrant women to participate. Even so, some of the women who were invited to take part in the study declined to participate.

In three interviews with women who were recruited in the waiting room of the free clinic, self-provided translators were used (persons who were already accompanying them as they were going to act as such in the medical consultation). These translators were informed about the importance of keeping confidentiality and discretion with the information treated during the interview.

In order to safeguard the anonymity of the participants, the researcher who collected the data transcribed and anonymised it before sharing it. Pseudonyms have been used to name the participants in this study.

Ethical approval for the study was obtained from the Ethics committee for research involving human subjects of the University of the Basque Country (UPV/EHU) before contacting any participant.

## Results

From the analysis of the data three categories were developed. Each category comprises structural barriers that hinder access of immigrant women to the health system and some facilitators that represent individual efforts to counteract these barriers.

The first category describes the factors that made women decide to avoid or to discontinue seeking healthcare, even in case of perceived need. The second category illustrates how miscommunication with professionals at the health centres compromised their access to appropriate care. The third category reflects the reasons expressed by the participants about how being immigrant and black influenced their access and the way they were (mis)treated in the health system.

### Fearing to enter a health system perceived as not friendly for immigrants

Reasons for avoiding or discontinuing to seek for healthcare were mainly related to fear of immigrants for being rejected, lack of knowledge on rights and health system organization and difficulty on fulfilling legal conditions and access procedures.

Fear of being rejected and considered undeserving healthcare attention based on their disadvantaged social position as immigrants was mentioned as an important barrier for avoiding asking for healthcare services. Likewise, participants expressed that the lack of knowledge about the laws regulating healthcare access as well as being undocumented acted as an additional strong reason for not approaching the healthcare centres or for approaching them with fear of being rejected.

As Maureen, an undocumented participant, stated about the influence of undocumentedness or the lack of lawful documentation for staying in the country:

*The first time to go to the health care centre for me, I was afraid. Ahh... my fear was... was that… that I would get there and the doctor will not attend to me. They could tell me: “Where is your document? You don't have any right to come here” (Maureen).*

Even if the Spanish law does not allow it, undocumented participants also avoided seeking care as they feared that the health system could report their personal data and undocumented status to state immigration institutions, which could lead to their deportation. Connected to such experience, participants also feared that in case of being asked for money to cover the expenses of the healthcare services, they could get into debt with a public institution, which could also contribute to their deportation.

*If you provide your personal details in the health centre, they can communicate to the Spanish government and they can take us out of the country. In addition, if you have no money… to incur a debt with the state can accelerate your deportation (Carol).*

Having the healthcare card was considered key for making rights going into effect, hence being able to support the access request with a lawful documentation and not being rejected. However, the main condition for being eligible to get it, being registered in the council register, was considered difficult to fulfil for those who were in a vulnerable economic and housing situation:

*To get the council registration you need to have money: If you have no money, you cannot pay for a room in a house where you can register as your permanent residence. And if you cannot get the registration, you cannot get the card… So you cannot access the health system (Gladis).*

The law regulating access to the health system, among other conditions, also establishes that any person can get healthcare services after payment. In the same way that misinformation prevented women to reach the health centres, being demanded to pay for receiving healthcare services, could also act as a discouraging factor to continue asking for healthcare, as Arjana pointed out:

*Imagine: I was very sick, but they were not attending me. The man there (administrative professional) told me: “Sorry, we can't do anything”. He also told me that I could pay and access, but I told him: “To pay? I have no money, no aid, I have nothing”. It was difficult. So I left (Arjana).*

Information about the process of getting access to health system is provided in the health centres. In this regard, participants noted that the administrative staff did not always provide complete and trustful information about immigrants' access legal requirements, needing to find other supporting resources, usually social organizations and the social network. To be informed or accompanied by someone who knew the legal access rights and the functioning of the health system, facilitated access. In addition, they pointed out that some professionals disobeyed the law and allowed their access even if they did not fulfil the legal conditions. However, the allowance was subject to individual factors, such as being known, being able to effectively communicate in Spanish or having insisted enough on the necessity to access.

*A nurse who knows me came to the counter and told the administrative professional: “Which is the problem with this woman?” And she said to me: “Come, come with me, we are going to see the physician and then we will think about how we can fix it in the program” (Teressa).*

In this category, we described how the participants' migrant status, together with the knowledge about legal rights and access procedures influence immigrants' access to healthcare services. However, these are not the only factors that determine their access, as they also need to overcome some communication barriers in the interaction with professionals, as the next category points out.

### Being attended on professionals´ own communication terms

Language was experienced as one of the most important barriers for accessing appropriate healthcare services. Consequences of the lack of communication between women and the different professionals in the health centres differed. With administrative staff, poor communication had a negative impact on their possibilities to meet a healthcare professional. With healthcare professionals, the impossibility to communicate hindered the possibility to be diagnosed and being offered a proper treatment, as Amara described:

*I have a problem. Now, I am in pain and I have gone to the physician some times. But as he could not understand me, I am still in pain. As I don't speak Spanish, the physician does not listen because he cannot understand anything and I just know how to speak French. And at the counter, it is also difficult to explain that you want to see a physician. It's complicated. Very complicated (Amara).*

In the same vein, miscommunication also led to mistakes in medical treatment that could result in harmful health consequences, as Arjana recalled:

*I took my cousin with me to the physician because my son was ill. The physician explained to him that the child needed to inhale the medication 3 times, with a space of 10 s. But my cousin told us that he needed to inhale it 10 times every 3 s. Imagine! To make him inhale, I needed to hold him, he couldn't breathe… I really thought that my son was going to die. I took him to the hospital and there we realized about the mistake (Arjana).*

Participants reported that administrative staff hardly ever spoke foreign languages, while healthcare professionals were found to be perceived to do it to a higher extent. However, they pointed out that professionals were not always committed to make efforts to communicate with them, alleging their poor consideration about immigrants as a possible reason.

*Where there is a reception, we are always poorly treated. That's like that. I have also experienced that. Some they don't even want to explain things to you. You are an immigrant, the behaviour… it is always bad towards immigrants (Amina).*

Women's inability to speak the local language was also perceived as putting them in a disadvantaged position, in which they were unable to answer to racist remarks from professionals or other users.

*Knowing the language, it is already an advantage. If people look at you with aversion, you can ask them: “Why do you look at me like that?” But if you cannot communicate… you are limited. You are afraid of facing them because you don't know what to say (Carol).*

However, not all experiences were negative. Participants appreciated the efforts of some professionals who tried to communicate with them and they described how sometimes both immigrants and professionals developed strategies to communicate, as acting by mimics or writing down and translating key information. Attending the health care centre with a translator was presented as a frequently utilised strategy by immigrants to better communicate. However, the use of self-provided translators, usually a friend, husband or descendant, was also described as a source of potential ethical conflicts related to confidentiality and privacy.

*Many people do not go to the health centre because of communication problems. Or if they go, they go with someone. But many times, you don't want the other person to know about your health problem (Jessy).*

The lack of an effective communication compromised not only the access of the immigrant women to healthcare services, but also their health. Additionally, the social consideration of immigrants and black people also played a role in their access and in the way they were treated in the health system, as the next category highlights.

### Is mistreatment based on racism or merely on bad luck?

Some participants expressed having received differentiated treatment or having been mistreated by professionals at the health centres. Administrative staff were considered a hard barrier to surpass to get healthcare attention. Differentiated treatment or mistreatment from their part was perceived in the way they behaved with immigrant women, compared to natives.

*At the counter, I usually pay attention to the way people are attended before me and I compare it with the treatment I receive. There is a huge difference between the way they treat natives and the way they treat me (Jessy).*

Meanwhile, healthcare professionals' vocation was widely considered as incompatible with being racist. Even if some situations of mistreatment from healthcare professionals were also reported, based on different cultural practices, their skin colour and professionals' stereotyped thoughts about immigrants. For example, Fatima presented the case on which the ignorance of the physician and the unwillingness to ask for clarification about a certain health practice, led to serious social and legal consequences for an immigrant woman she knew.

*A women from Mali went to the paediatrician and he told her to undress the baby. We always spread shea butter on the babies. And it stinks, smells like cheese, but it is good for their health. The physician directly called the social worker: “Look, there is a woman in the consultation who does not take care of the child. The child stinks”. Then, she had many-many problems because they wanted to take her child out from her (Fatima).*

Amina reported how she felt bad to be unnecessarily characterized as a black woman in the clinical record. What is particularly interesting from her quote is how Amina, despite feeling bad about this and perceiving a differentiated treatment towards her, she stressed that the health care professionals' behaviour was good.

*The physicians wrote in my report: “A black woman, or black raced, which such and such symptom…” And I told him: “Why do you write that?” That made me feel a bit badly. Their behaviour was good! But even if they didn't tell me anything, I could feel that something was going on with me (Amina).*

Some cases on which healthcare professionals' stereotyped thoughts on immigrants also influenced the diagnoses were presented. For example, Amina related that a few years ago, while she was admitted at the hospital, she suspected that professionals were afraid of approaching her as they were considering her to be a possible Ebola case patient, when she had not even left the Basque Country in the last years. Likewise, Carol experienced that the physician did not want to request for medical tests, stating that her symptoms were based on the consequences of her migration process, and a lack of acclimation to the host country.

*I explained to the physician that I have been ill for two months: I had the period, period, period… but she was just telling me that it was because I was not acclimated here. I understood that she was using an excuse not to treat me. But it was not like that, I was seriously ill! (Carol).*

Being asked about the reasons of this perceived differentiated treatment for immigrants in the health system, two participants who worked at social organizations, Amina and Mariam, made a connexion between the poorer attention given to black people in the health system (same as in other institutions and in society) with historical colonial domination of high-income countries. They stated that the image of Africa presented in the mass media as a homogeneous land of corruption, poverty and famine reinforces a negative collective social imaginary on Africans and black people shared by the general population and by professionals working at the health system, which leads to a differentiated treatment and hinders access.

*People associate not speaking the language and not knowing anything with the black colour, and there is an imaginary that blacks are like animals, uneducated, rude, primary, ignorant… All those labels that the history has accumulated on us influence the way we are attended. A social rejection imaginary follows us wherever we go and hinders our access to the health system (Mariam).*

They also described how professionals consider immigrants as over demanding and undeserving social and economic public resources.

Lastly, the colonial history was also pointed to be responsible for the internalized sense of inferiority felt by black immigrants, making it difficult for them to stand for their rights on host countries. Consequently, they felt illegitimate to ask for social benefits and public services, and even to a greater extent when they are undocumented.

*Black people have the fear to be rejected internalized, after too many years of colonization and slavery. And now the immigration is another extension of them. Therefore, they have already internalized that they are inferior, as such, they take for granted that they have no value (Mariam).*

While Amina and Mariam connected the way immigrants were (mis)treated with colonialism and racism, other participants described the cases of mistreatment as anecdotal, not exclusively happening to black immigrants and based on the professional's bad day, more than on systemic discrimination.

*I think that you (as black immigrant) are treated like any other person. It is different if you go one day and there is a person in the bad mood who treats everyone badly, but that can happen to anyone (Anne Marie).*

Other participants pointed specifically out that having black skin colour was not the reason for a possible poorer attention or mistreatment, while they could also recognize that some professionals provide a differentiated medical attention based on it.

*There are mistakes from professionals, but they have nothing to do with the skin colour. No, no, no, no, due to colour no, I've never found that. I know some that they experienced it, but not me. They say: “the physician could at least touch me!” They don't touch you. I used to have a physician who didn't feel repulsion for us: she used to touch us and then, she was washing her hands (Precious).*

Likewise, the presumed ignorance of the healthcare professionals about black people which led to disrespectful treatment, was described at some point as understandable, anecdotal and a justifier for having received a differentiated treatment being a black woman.

*When I was pregnant, my gynaecologist told me that he was curious about how was the labour of a black woman. And when I was operated, a surgeon told me that he had never seen a black inside. It is an understandable anecdote based on the ignorance that any of us can have about the others (Teressa).*

## Discussion

Our findings suggest that Sub-Saharan African immigrant women need to overcome numerous barriers to gain access to appropriate healthcare in the Basque health system. This study also highlights the crucial role of structural racism in generating and sustaining such barriers: the third category represents the influence of a racist shared social imaginary of immigrants and black people regarding their access to appropriate healthcare, which also reinforces the barriers in the first and second categories. That is, these shared stereotyped preconceptions negatively affect the willingness of the health systems and their staff to be organised in a way that is responsive to the needs of a culturally, linguistically and socially diverse population.

In most countries, policies establish different entitlements to healthcare services depending on the origin, social conditions and migration status [37]. This poses a challenge to the right to health declaration that states that all human beings are entitled to the highest attainable standard of health [38]. Such policies contribute to create health inequities by generating, within the same country, different categories of entitlements for different populations. Vulnerable populations, such as undocumented immigrants and asylum seekers, are many times considered undeserving of those services and perceived as an economic burden. In consequence, their entitlement to healthcare is conceptualized as a benevolent action or a privilege rather than an exercise of their rights [8, 39–41].

As our study shows, the social vulnerability of undocumented immigrants, induced by their legal situation, made it more difficult for them to get a source of income and to pay for accommodation to be registered as a resident [8, 39], which is a sine qua non condition to get the healthcare card in Spain. In addition, undocumented immigrants suffer from stigma on the basis of documentation status [24] and as a result, experience more problems than the documented ones to access healthcare, for fearing to be rejected by the health centre professionals or reported to immigration authorities [1, 5, 6, 8, 9]. Our findings align with previous studies that show that access for undocumented immigrants is unequal and strongly dependent on the goodwill of individual professionals [5, 8]. Thus, undocumentedness acts as a great barrier not only for immigrants' entitlement to the right to healthcare, but for maintaining good health and mental health outcomes [42].

Similarly to our results, previous studies have shown that the lack of entitlement to the right to healthcare is an important barrier for immigrants to access healthcare [2, 8]. The complexity of administrative procedures, such as obtaining the healthcare card in the Spanish NHS and the demand for services payment, are also considered in the literature as difficult to understand and to follow for immigrants [6, 40], preventing them to access healthcare [5, 6, 8, 39, 43]. Contrary to immigrants' reported experiences, healthcare workers and managers in other Spanish regions have not perceived immigrants to have any difficulty or barrier in the process of getting the healthcare card [44, 45]. However, professionals working at free clinics in the Basque Country have expressed their concern about the difficulties on accessing public healthcare services by immigrants [2]. Like in our participants' narratives, lack of awareness of legal entitlements among both, immigrants and the staff at the health centres, has also been extensively described in other studies as an important access barrier for immigrants [5, 6, 8, 39], resulting in avoiding to access or receiving mislead information about their rights at the first point of contact with health services [8, 40].

In addition to entitlement related obstacles, the language barrier that this study highlights has also been extensively described in the literature as one of the main challenges to an effective access to healthcare for immigrants [5, 6, 8, 39, 40, 43, 46]. In our setting, telephone based translation service is available in the health centres, even if the legal norms do not specifically state the right of patients for being provided a translator. The unwillingness of professionals to communicate or make use of translating tools has to be contextualized within a system that i.e. does not reward for the knowledge of non-local languages to non-healthcare staff (like administrative professionals) and pressure healthcare professionals to attend large number of care-seekers through short consultations.

It is known that ineffective communication may compromise care, but there are also important factors that can favour the communication and relationship between patients and professionals, like a receptive attitude or the efforts made by professionals to meet patients' necessities and to understand their cultural characteristics [46]. In contrast, the lack of cross cultural knowledge complicate healthcare providers' understanding of the reasons for consultation and the therapeutic approach to be undertaken [47]. Even if using interpreters (usually family or friend) is a usual strategy for immigrants to communicate in the health system, their use does not mean that patients' needs and concerns are heard [8, 46]. On top of that, the use of inadequate or non-professional interpreters can lead to ethical problems about confidentiality and privacy [39].

While legal barriers and communication challenges have been extensively explained in the literature, the role of the racism has not been clarified in previous studies on access to healthcare and migrant populations. Structural racism as manifest in ideologies, practices, processes and institutions that operate to produce and reproduce differential access to power [13] is deeply enrooted in social institutions, including the health system, its culture and norms, and thus it is not surprising that it also permeates the professional-patient interaction [48]. In general, health systems in Spain are organized “for a culturally, linguistically and socially uniform population”, creating inequality in access and use of healthcare services [49].

The influence of structural racism in healthcare access and health remains usually invisible, but perceived discrimination is associated with lower levels of physical and mental health and poorer access to healthcare [50, 51]. Even if it has been repeatedly stated that immigrants use the healthcare services to a lesser extent than the native population [21, 22, 52, 53], health workers usually share the opposite vision. Consequently, access disparities, delays or avoidance of accessing healthcare and mistrust of the health system among immigrants increase [48, 54]. Especially in times of crisis, immigrants have been pointed as “over users” of healthcare services [44], reinforcing the discourse that constructs migrants as an “other” who underserves healthcare and social benefits [40]. Same as this study identified, a study in France revealed how the caregivers also projected inherited representations of colonialism on Africans, marking them either as violent or as naive, which provoke differentiated healthcare practices, and also affected the way they were attended and treated [18].

The paradox of expressing overall satisfaction with healthcare services despite of having perceived discrimination or bad treatment that arose in our study was also present in a research about immigrants' perceptions of healthcare attention in the Spanish NHS. While almost all the participants expressed having been treated respectfully, at the same time, 60% also perceived discrimination towards immigrants and almost 70% answered that cultural differences negatively affected the quality of received attention [55]. Lack of trust on the health systems in the origin country contributes to having a good consideration of the health system at the host country despite of perceiving discrimination in attention [43, 44]. Likewise, reconstructing experiences of discrimination as random and not based on racism, as appeared in our results, can also be a copying strategy for “taking over” the consequences derived from those situations, such as accepting being oneself in a disadvantaged social position [56].

The health system organization and the health workers' practices can contribute both to preserving or transforming the power relations within the health systems [19]. Even if several barriers coming from the staff were identified, some facilitators, mainly related to individual professionals' good practices were also found, such as attempts for changing what they considered an unfair situation. When healthcare rights were reduced in 2012, a number of strong healthcare professionals' networks were created calling to disobey the new restrictive legal conditions and to respect universal health coverage [57]. Moreover, healthcare professionals' ethical guidelines, such as deontological codes, go beyond the changeable content of the laws, as they promulgate the necessity of attending all patients with the same diligence, without discriminating against any person.

### Methodological considerations

The present study contains certain methodological strengths and weaknesses that should be mentioned.

The researchers' social position, cultural standard, personal characteristics and experiences shape the research process [58]. In this study, none of the authors have Sub-Saharan origin nor are immigrant in the Spanish context, so they had an outsider-perspective on the topic, which has advantages and disadvantages for the research process and outcomes. As the authors had not experienced the studied topic, the participants were considered to be in expert positions, which could be part of an empowering process for them. Likewise, the researchers approached the topic with an open viewpoint that may lead to identifying disguised associations and considering the topic from a broader perspective than the experiential. However, the absence of insider perspective may also lead to inability to fully comprehend the participants' experience or reflect it appropriately.

Some language nuances may have been missed during the interviews or during translation of the interviews from French to Spanish. However, offering the participants the opportunity to conduct the interview in the language they preferred, so they could better explain themselves, is also a strength of the study.

Our study explains the influence of structural racism on the received treatment and barriers to appropriate healthcare attention from a qualitative perspective. Future studies should deepen the study of the role that racism plays in healthcare attention and how it affects vulnerable populations' health indicators and decisions to access the health system.

## Conclusions

For Sub-Saharan African immigrant women, accessing appropriate healthcare in the Basque Country was perceived to be challenged by institutional barriers. Structural racism compromises the provision of an appropriate access to healthcare as it guides access legal conditions, health system organization and social ideologies, which are also present on practices of the staff at health centres.

At legal level, barriers included lack of entitlement, difficult fulfilment of legal conditions and the negative influence of being undocumented for accessing. Negative social imaginary on immigrants and black people, usually shared by staff at the health centres, was in the base of discriminatory treatment they received. During individual encounters, lack of an effective communication with staff at the health centres compromised not only the access but their health. Meanwhile, several facilitators coming from individual professionals and social actors who eased their access providing information, accompaniment and support were identified.

Reinforcing non-discrimination values and addressing the importance of providing a culturally appropriate and rights based attention in the curricula, paying special attention on avoiding racist behaviours, the use of cultural mediation and having more approachable sources of information, would be key to get a more inclusive and non-discriminatory health systems.
